# Physicochemical and Microstructural Characterization of Whey Protein Films Formed with Oxidized Ferulic/Tannic Acids

**DOI:** 10.3390/foods10071599

**Published:** 2021-07-09

**Authors:** Yaosong Wang, Youling L. Xiong

**Affiliations:** 1 Department of Food Science and Engineering, College of Light Industry and Food Engineering, Nanjing Forestry University, Nanjing 210037, China; yaosongwang@njfu.edu.cn; 2 State Key Laboratory of Food Science and Technology, Jiangnan University, Wuxi 214122, China; 3 Department of Animal and Food Sciences, University of Kentucky, Lexington, KY 40546, USA

**Keywords:** whey protein, edible film, oxidized polyphenols, crosslinking, morphology

## Abstract

Protein-based biodegradable packaging films are of environmental significance. The effect of oxidized ferulic acid (OFA)/tannic acid (OTA) on the crosslinking and film-forming properties of whey protein isolate (WPI) was investigated. Both of the oxidized acids induced protein oxidation and promoted WPI crosslinking through the actions of quinone carbonyl and protein sulfhydryl, and amino groups. OTA enhanced the tensile strength (from 4.5 MPa to max 6.7 MPa) and stiffness (from 215 MPa to max 376 MPa) of the WPI film, whereas OFA significantly increased the elongation at break. The water absorption capability and heat resistance of the films were greatly improved by the addition of OTA. Due to the original color of OTA, the incorporation of OTA significantly reduced light transmittance of the WPI film (λ 200–600 nm) as well as the transparency, whereas no significant changes were induced by the OFA treatment. Higher concentrations of OTA reduced the in vitro digestibility of the WPI film, while the addition of OFA had no significant effect. Overall, these two oxidized polyphenols promoted the crosslinking of WPI and modified the film properties, with OTA showing an overall stronger efficacy than OFA due to more functional groups available.

## 1. Introduction

Biodegradable packaging materials are being developed as a part of “future food” to partially replace traditional nondegradable materials, to reduce environmental pollution [1]. Of the different biodegradable materials, whey protein has received considerable attention, due to its film-forming potential [2]. Whey protein is capable of forming a barrier film with high transparency, a uniform texture, and flexibility [3,4]. However, unlike plastic packaging materials made of polymerized organic molecules, edible films made from native proteins lack structural integrity and mechanical strength [5,6]. To mitigate the deficiency, methods that are capable of modifying the reactive side-chain groups in proteins are recommended, i.e., physical, chemical, or enzymatic treatments [7].

Protein crosslinking is considered to be an effective approach in achieving desirable functionality for film production [8]. Suitable crosslinkers include a variety of natural compounds that prove to be safe and present minimal risk. Phenolics, one of such compounds that are widely available, are a large class of non-toxic, antioxidative, and edible natural products, of which many exhibit health benefits [9]. In food processing, polyphenols are readily oxidized to electrophilic quinones, which can subsequently crosslink with proteins [10]. Previous studies have shown that ferulic and tannic acids, two widely distributed natural phenols, have antimicrobial and antioxidant activity [11,12]. Both also have the potential to improve the mechanical properties and decrease the water vapor permeability of collagen- or gelatin-based films [13,14]. Oxidized tannic acid also strongly affects the mechanical strength, surface hydrophobicity, and hydration of zein-based films [15]. Ferulic acid is capable of decreasing the oxygen permeability of whey protein films [4], while tannic acid is able to crosslink caseins [16] and β-lactoglobulin (βLg) [17], leading to significantly improved overall physicochemical properties of such edible film products.

Due to the electrophilic nature of quinone carbonyls and the abundant presence of nucleophiles in whey protein (sulfhydryl and amine groups), it can be hypothesized that the quinone species derived from oxidized ferulic or tannic acids are more efficient to crosslink whey proteins to produce functional protein films. To the best of our knowledge, few studies have been conducted to investigate the impact of oxidized phenolic acids on the crosslinking of whey protein in edible film production. In this context, the objective of our resent work was to employ oxidatively modified ferulic/tannic acids to crosslink whey protein for an enhanced film with improved physicochemical properties. The digestive performance and microstructure of the resulting films were also investigated.

## 2. Materials and Methods

### 2.1. Materials and Chemicals

Whey protein isolate (WPI, 90% protein) was obtained from Hilmar Ingredients (Hilmar, CA, USA). Pepsin and pancreatin (8 × USP) were purchased from Sigma-Aldrich, Inc. (St. Louis, MO, USA). Ferulic acid (C_10_H_10_O_4_, a monophenol with a molecular mass of 194.2) and tannic acid (C_76_H_52_H_46_, a decagalloyl or decaphenol with a molecular mass of 1701.2) were purchased from Sinopharm Chemical Reagent Co. Ltd. (Shanghai, China). All other chemicals and reagents, of analytical grade unless otherwise specified, were purchased from Sigma-Aldrich (St. Louis, MO, USA) or Sinopharm Chemical Reagent Co. Ltd. (Shanghai, China).

### 2.2. Oxidation Phenolic Acids

The oxidized ferulic and tannic acids were prepared according to the method described in a previous study [18] with slight modifications. Ferulic and tannic acids (1.125 g) were dissolved in 45 mL deionized water at 60 °C with gentle stirring. The solutions were adjusted to pH 9.0 with 1 N NaOH and then incubated in a 50 °C water bath. The solutions were bubbled with oxygen during the reaction for up to 3.5 h to convert both acids to quinones.

### 2.3. Film-Forming Solutions Preparation and Film Casting

Aqueous WPI solutions (6%, *w/v*) were adjusted to pH 8.0 using 1 M NaOH and then heated at 90 °C for 30 min in a water bath to unfold protein. After cooling to room temperature (23 °C), prepared phenolic compounds (0, 2.5, and 5.0% *w/w*, protein weight basis) were added. The mixtures were stirred at room temperature for 1 h, and the plasticizer glycerol (2.4% *w/v*, final concentration) was then incorporated and gently mixed. Prior to cast, the solutions were degassed under vacuum for 10 min to remove air. Aliquots were poured onto leveled plexiglass plates and spread evenly with a glass rod [3]. Films were dried in a humidity-controlled chamber (PGC Parameter Generation & Control, Inc., Black Mountain, NC, USA) with a 50 ± 2% relative humidity (RH) at 23 °C for 24 h and then peeled for subsequent evaluation.

### 2.4. Measurement of Protein Changes

#### 2.4.1. Total Sulfhydryl Content and Free Amines

Total sulfhydryls (SH) were measured using 5,5′-dithiobis (2-nitrobenzoic acid) (DTNB) as previously described [19]. The absorbance at 412 nm was measured against a blank of DTNB at the same concentration without protein. Total sulfhydryl concentration was calculated using a molar absorption coefficient of 13,600 M^−1^ cm^−1^ and expressed as nmol sulfhydryls per mg protein. Free amines (NH_2_) were quantified according to the colorimetric reaction with trinitrobenzenesulfonic acid (TNBS) [20]. WPI samples were diluted to 1% in 2.0 mL of 0.2 M phosphate buffer (pH 8.2), and 1.0 mL of 0.01% TNBS solution was added. The mixture was incubated at 50 °C for 30 min in the dark before 2 mL of 0.1 M sodium sulfite was added to terminate the reaction. Absorbance at 420 nm was then measured, and the free-amine content was calculated as µmol amines per mg protein using a standard curve constructed from L-leucine.

#### 2.4.2. Surface Hydrophobicity

Surface hydrophobicity as an indicator of protein reactive nonpolar group exposure was determined using a fluorescence probe, 8-anilino-1-naphthalene sulfonic acid (ANS) [21]. WPI samples were diluted to 10 mg/mL, and 4 mL of the solution was reacted with 0.4 mL of 0.04% ANS for 15 min at room temperature. Fluorescence intensity was measured using a Hitachi 650–60 fluorescence spectrophotometer (Hitachi Ltd., Tokyo, Japan) with an excitation wavelength of 365 nm and emission wavelength of 470 nm.

#### 2.4.3. ζ-Potential and Particle Size

A Mastersizer Nano ZS instrument (Malvern Instruments, Worcestershire, UK) was used to measure ζ–potential and particle size distribution of WPI cast solutions. The protein concentration was diluted to 1 mg/mL using 10 mM phosphates buffer (pH 7.0). A 1 mL sample was used to measure the particle size and ζ-potential at 25 °C after 2 min quiescent equilibrium post sampling. The values of ζ–potential and mean diameter Z-average were expressed as the average of three freshly prepared cast solution samples.

#### 2.4.4. Sodium Dodecyl-Sulfate Polyacrylamide Gel Electrophoresis (SDS–PAGE)

Electrophoresis was performed according to the method of Laemmli [22] using a 5% acrylamide stacking gel and 12.5% acrylamide separating gel. The gel was stained with 0.1% (m/v) Coomassie brilliant blue (R250) in 50% methanol and 6.8% glacial acetic acid solution for 4 h and subsequently destained with a 7.5% glacial acetic acid and 10% methanol solution.

### 2.5. Assessment of Film Properties

#### 2.5.1. Mechanical Properties

Prior to measurement, films were conditioned in the humidity-controlled chamber (RH 50 ± 2%) at 23 °C. A TA.XT plus texture analyzer (Stable Micro Systems Ltd., Godalming, UK) was used to determine tensile strength (TS), elongation at break (EAB), and Young’s modulus according to ASTM [23] standard method. Samples were prepared by cutting into 1.9 cm × 10 cm strips using a sharp knife and then mounted to the texture analyzer. A 50 N load cell was used to test the films at a pull rate of 50 mm/min. TS and EAB were calculated from the curve of the force versus distance.

#### 2.5.2. Swelling

Preconditioned (23 °C, 50 ± 2% RH) films were cut into 2.5 × 2.5 cm^2^ squares and immersed in 100 mL of warm (40 °C) deionized water for 30 min. After blotting with filter paper, the film samples were weighed. The degree of swelling was calculated according to Equation (1):

(1) $$S\;\left(\%\right)=\frac{W_{2}-W_{1}}{W_{1}}\times 100\%$$

where *W*_1_ and *W*_2_ are the original and swollen weights, respectively.

#### 2.5.3. Water Vapor Permeability (WVP)

WVP was determined gravimetrically according to ASTM [24]. Films were mechanically mounted onto a beaker (height: 6 cm; d = 3.3 cm) filled with silica gel at 5 cm of depth. The beakers were placed in the 50% RH chamber at 23 °C. The weight of beakers was measured at 2 h intervals for a total of 12 h. WVP was calculated according to Equation (2):

(2) $$WVP=\frac{W\times x}{t\times A\times \Delta p}$$

where *W* is the weight gain within 12 h (*t*), *x* is the film thickness (mm), *A* is the area of exposed films (m^2^), and Δ*p* is the difference in vapor pressure across the film equal to 1.4052 × 10^3^ Pa.

#### 2.5.4. Microstructure and Morphology

The fine structure of film cross-sections was characterized by using scanning electron microscopy (SEM, Quanta-200 scanning electron microscope, Hillsboro, OR, USA). The film was mounted on a bronze stub and sputter-coated with gold prior to imaging.

For film surface morphology characterization, atomic force microscopy (AFM) was conducted using an MFP-3D-SA AFM machine (Asylum Research, Goleta, CA, USA) in the tapping mode. Roughness (*Rq*) was calculated according to Equation (3):

(3) $$R_{q}=\sqrt{\frac{\sum_{i=1}^{N}{\left(Z_{i}-\bar{Z}\right)}^{2}}{N}}\;\mathrm{and}\;\bar{Z}=\frac{1}{N}\sum_{i=1}^{N}Z_{i}$$

where *Z_i_* is the height of values profile (histogram, nm), $\bar{Z}$ is the arithmetic mean of heights (nm), and *N* is the number of data points in the profile.

#### 2.5.5. Light Transmittance and Transparency

The ultraviolet (UV) and visible-light barrier properties of WPI films were measured at 200–800 nm using a UV–visible spectrophotometer (Gary50, Varian, Palo Alto, CA, USA). Film transparency was calculated as in Equation (4):

(4) $$T=-\log T_{600}/x$$

where *T*_600_ is the fractional transmittance at 600 nm, and *x* is the film thickness (mm).

#### 2.5.6. Thermal Properties

Differential scanning calorimetry (DSC) was conducted to determine the thermal transition temperatures of the films [25] using a Q2000 DSC machine (TA Instruments, New Castle, DE, USA). Approximately 3 mg of film samples were placed in an aluminum pan and hermitically sealed. Samples were heated from 25 °C to 260 °C at a rate of 20 °C per min. An empty sample pan was used as reference. The transition temperatures (onset, *T*_0_; peak, *T*_p_; and end, *T*_d_) and enthalpy of melting (Δ*H*) were determined from the thermograms using Universal Analysis 2000 software (v. 4.5A, Build 4.5.0.5) supplied by TA Instruments (New Castle, DE, USA).

#### 2.5.7. Film Protein Leachability

Films were trimmed into 2.0 × 2.4 cm strips, individually submerged in 10 mL of McIlvaine buffer (50 mmol/L, pH 3–7), and placed on a platform shaker at room temperature for 24 h. The leached proteins were quantified by the Biuret method. The leachability was expressed as the ratio of protein released into the solution versus the total protein in the original film. The supernatant containing leached proteins was also subjected to SDS–PAGE analysis.

#### 2.5.8. Film Digestibility

Proteolytic digestion of the films was performed using gastrointestinal enzymes [26]. Aliquots (0.2 g) of ground film pieces were transferred into 100 mL Erlenmeyer flasks to which 50 mL of deionized water was added. After adjusting the pH to 2.0 using 1 N HCl, the film samples were incubated at 37 °C for 60 min then centrifuged at 5000× *g* for 15 min. Leached protein (supernatant) was measured, and unleached protein (%) was calculated as follows: [(total film protein–leached protein)/total film protein] × 100. Pepsin (4%, *w/w*, protein basis) was then added. The mixture was incubated 1 h in a shaking water bath at 37 °C. Subsequently, the gastric digest was adjusted to pH 7.5 by the addition of 0.9 M NaHCO_3_, and pancreatin (4% *w/w*, protein basis) was then added. The solutions were incubated at 37 °C for up to 4 h. At 0, 30, 60, 90, 120, 180, and 240 min, 2.5 mL aliquots were taken and 1 mL of 50% (*w/w*) trichloroacetic acid (TCA) was added to terminate the digestion. All inactivated samples were stored at 4 °C for 18 h and then centrifuged at 5000× *g* for 15 min. The protein (peptide) concentration of the supernatant was determined by the Biuret method. The percentage of enzyme-released protein over the total amount of unleached protein (i.e., remaining in the film prior to pepsin addition) was designated as the digestibility or digestion rate (%).

### 2.6. Statistical Analysis

The experiments were conducted in triplicate, each with at least duplicate sample analyses. Data were subjected to the analysis of variance using the Statistix software 9.0 (Analytical Software, Tallahassee, FL, USA) adopting a general linear model procedure. Significant (*p* < 0.05) differences between means were identified by LSD all-pairwise multiple comparisons.

## 3. Results and Discussion

### 3.1. Protein Modification and Physicochemical Changes of Film-Forming Solutions

Susceptible to reactive oxygen species (ROS), protein side-chain groups are an easy target, and serve as the basis for crosslinking reactions with polyphenols under oxidative conditions [27,28]. In particular, sulfhydryl, free-amine, indolyl, and imidazolyl groups are involved in covalent reactions between proteins and phenolic compounds [29]. In the present study, oxidized ferulic acid (OFA) and, more so, oxidized tannic acid (OTA) showed a major, dose-dependent effect on sulfhydryls and free amines (Table 1). The loss of the two protein side-chain groups ranged from 24.7 to 94.7 for SH, and from 5.1 to 25.3% for NH_2_ (*p* < 0.05). The most likely cause was electrophilic attack by both the quinone species. While OFA did not affect protein surface hydrophobicity, OTA significantly decreased the value, due to the induction of protein aggregation, which was evinced by the marked increase in protein particle size (Table 1). The stronger efficacy of OTA was attributed to the polygalloyl structure (10 phenol groups) of tannic acid compared with only one phenol in ferulic acid; hence, the potential of generating highly reactive polycarbonyls when oxidized. The particle size increase can be caused by a number of forces promoted by the presence of oxidized phenolic compounds, including disulfide linkages, hydrogen bonds, and van der Waals force, as well as protein–phenol complexation [10]. These physicochemical modifications did not have a significant effect on protein charge distribution (ζ-potential). The structural changes and crosslinking reactions induced by oxidized phenolic acids would conceivably modulate the film-forming ability of WPI.

**Figure 1:**
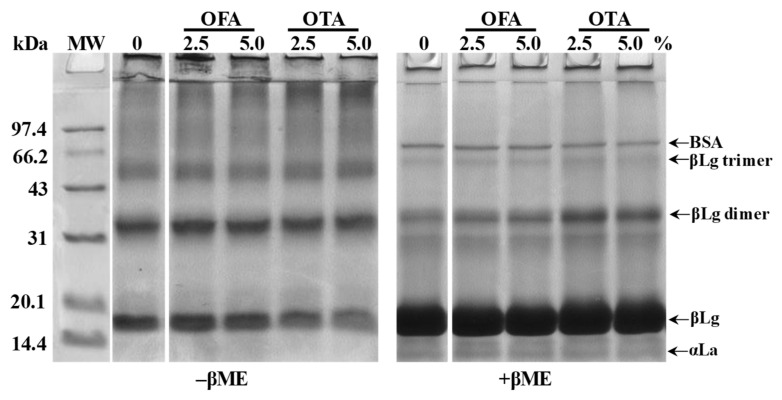
Sodium dodecyl-sulfate polyacrylamide gel electrophoresis (SDS–PAGE) of whey protein isolate (WPI) treated with 2.5% and 5.0% oxidized ferulic (OFA) and tannic (OTA) acids under non-reducing (−βME) and reducing (+βME) conditions. MW: molecular weight; BSA: bovine serum albumin; βLg: β-lactoglobulin; αLa: α-lactalbumin; βME: β-mercaptoethanol.

To detect covalent cross-linkages between the protein molecules, caused by the oxidized phenols, electrophoresis under reducing (with β-mercaptoethanol, +βME), and non-reducing (without β-mercaptoethanol, −βME) conditions was performed (Figure 1). All the WPI samples, regardless of phenol treatment, displayed a prominent βLg band and its dimer. However, protein polymers accumulating as smears, near the top of the separating gel, were ostensibly more intense in oxidized WPI than in the control sample, indicating covalent crosslinking. The oxidative effect of OFA and OTA differed considerably. For the samples treated with OFA, there were no remarkable changes in the protein pattern (−βME). Although largely broken down under reducing conditions (+βME), the βLg dimer remained salient in the OFA-treated samples, suggesting secondary crosslinking via quinones other than the primary disulfide bonds. These electrophoretic changes were more noticeably pronounced when WPI was treated by OTA, which included the disappearance of βLg, OTA-crosslinked βLg dimer, and the formation of protein polymers. Previous studies had shown that phenols and their oxidized quinone forms could react with proteins via C–N or C–S bonds [30,31]. Our results, in support of those previous reports, were also consistent with the observed decline in both the SH and the NH_2_ groups (Table 1).

### 3.2. Mechanical Properties

Tensile strength (TS), elongation at break (EAB), and Young’s modulus (modulus of elasticity) are commonly used to describe the mechanical properties and structural integrity of protein films [5]. These parameters are considered in the handling, processing, and storage of films when applied to practical foods. The addition of oxidized phenolics significantly improved TS and Young’s modulus, especially by OTA (*p* < 0.05), and there was no difference between the 2.5% and 5.0% dosage levels (Table 2). While EAB was greatly increased by OFA, suggesting a strong film elasticity, it was not significantly affected by OTA. The inherent changes in a molecule, such as polypeptide stretching [32] and the hydrophobicity of phenol-bound proteins [33], could affect the mechanical properties. Oxidized phenolics can induce protein aggregation and react with proteins via nucleophilic pathways to form C–N or C–S through quinone carbonyls, leading to protein crosslinking [30]. These changes likely accounted for the observed improvements in the mechanical properties of the oxidized WPI films in our study.

### 3.3. Swelling and WVP

The water absorption capacity of the WPI film, expressed as the degree of swelling, increased modestly with OFA, but remarkably (>2.3-fold, *p* < 0.05) by OTA (Table 2). It appears that an equilibrium level was already reached with 2.5% phenolics, as no additional effect was produced at the higher concentration (5.0%). The changes may be related to the microstructure of the film matrix, such as porosity, and the hydrophilicity of the protein material after crosslinking [34]. Although both of the oxidized phenols promoted protein crosslinking, this modification had no measurable effect on water vapor permeability (WVP), due to the hydrophilic nature of the WPI film, which is similar to the finding reported previously [35]. The arrangement of protein molecules, film microstructure, and plasticizers (e.g., glycerol) also affected the WVP. It is noteworthy that the comparison of phenol treatment effects in the present study was made under a specialized condition (50% RH at 23 °C and 12 h). The values obtained may be subject to change under other RH conditions, since the water vapor transmission rate may be affected.

### 3.4. Microstructure

**Figure 2:**
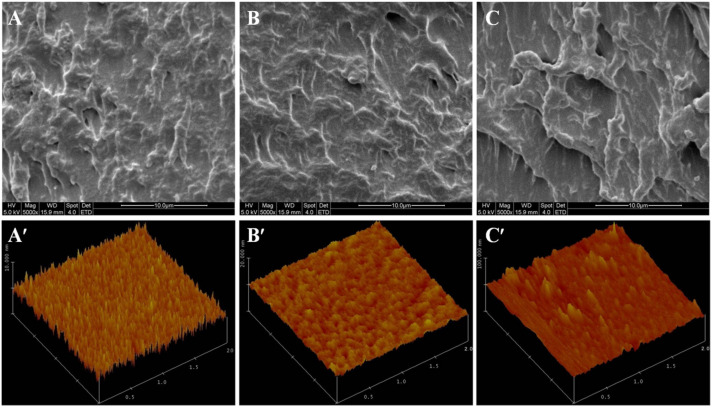
Cross-section SEM images (upper panel) and AFM surface images (lower panel) of control (**A**,**A′**), 2.5% oxidized ferulic (OFA; **B**,**B′**) and tannic (OTA; **C**,**C′**) acid-treated WPI films.

To explain the influence of OFA and OTA on the functional properties of WPI films, the interior and surface structures of the prepared films were examined by scanning electron microscopy (SEM) and atomic force microscopy (AFM), respectively. The cross-sectional SEM micrograph of the control WPI film was relatively orderly and uniform (Figure 2A); the OFA-treated WPI film appears to be more delicate, with continuous protein strands (Figure 2B), while the OTA treatment caused protrusions, ridges, and pores in the film (Figure 2C). These structural variations may reflect the crosslinking behavior of oxidized phenols, and explain the enhanced mechanical (TS, EBA, and Young’s modulus) and water absorption properties (Table 2). The structure–functionality relationship of protein-based films has also been reported in other studies, for example, polysaccharide-included whey protein films [36].

Furthermore, the morphological images of the WPI films, acquired by AFM, exhibited considerable differences due to oxidized phenol treatments. Remarkably, the maximum height scale of the protein aggregates in the 2.5% OTA-treated WPI film (Figure 2C′) increased from 5 nm (control film; Figure 2A′) to 15 nm (*p* < 0.05). Meanwhile, the aggregates became rougher and more protruding. Other researchers also found that the addition of oxidized sinapic acid resulted in more clustered areas compared to the pure WPI film [37]. On the other hand, for the WPI film treated with OFA (Figure 2B′), despite the slightly increased roughness, the maximum height of the aggregates remained at approximately 5 nm. These morphological changes were due to the further aggregation of oxidized polyphenols in the film formation process, after the crosslinking of the WPI. The AFM results corroborated with SDS–PAGE, which showed markedly increased crosslinking of WPI in the presence of OTA and moderate crosslinking caused by OFA (Figure 1).

### 3.5. Light Transmission and Transparency

Apart from preventing gas exchange (O_2_, CO_2_, water vapor, etc.), blocking the absorption of light is also an important property of edible films. As shown in Table 3, all of the WPI films resisted penetration by UV light (200 and 280 nm). However, the OFA- and OTA-treated films had markedly reduced light transmission at 350 and 400 nm, and the efficacy was phenolic dose-dependent, i.e., the higher the concentration oxidized phenols, the stronger the blocking effect. In the wavelength of 500–800 nm, the film formed with the crosslinking OTA still showed a respectable light-blocking effect, while the effect of OFA was poor. Film transparency decreased at any OTA concentration, while OFA had no effect. Due to the presence of oxidized polyphenols, the OTA-treated WPI film was darker than the OFA-treated film (brown). These films could be potentially used to package photosensitive foods, such as those rich in unsaturated fatty acids and susceptible to pigmentation, thus fulfilling the diversity requirements for food packaging materials [6].

### 3.6. Thermal Properties

**Figure 3:**
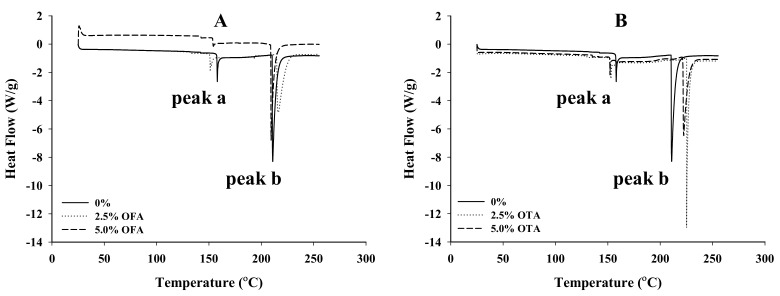
Differential scanning calorimetry thermograms of 2.5% and 5.0% oxidized ferulic (OFA; **A**) and tannic (OTA; **B**) acid-treated WPI films.

The thermal melting profile of protein matrix films is an important property for industrial applications (e.g., as edible pouches for instant food and dry ingredients), where heat resistance and sealing strength must be considered [38]; the glass transition temperature of the protein material is one of the determinant factors [39]. Figure 3 displays the DSC melting profiles of the control and oxidized phenol-crosslinked WPI films that were subjected to heating from 25 to 260 °C. The thermal curves can be divided into two temperature zones according to rate of heat input. A small endotherm was recorded immediately above 150 °C (peak a), which can be attributed to the molecular transformation of weak interaction forces or low-order structures [40], including the melting of the partially crystalline/amorphous whey protein [41]. The peak was attenuated in OFA- and OTA-crosslinked WPI films when compared with the control, suggesting a stronger water-binding capacity in the phenol-crosslinked films. A major endothermic transition occurred in the temperature region of 210–240 °C (peak b). The destruction of ordered molecular structures (e.g., hydrogen bonds) and the possible thermal decomposition of polypeptide chains, along with the volatilization of glycerol, may be responsible for this transition [42,43]. While the effect of OFA on this melting temperature was small (2–5 °C, Figure 3A), that of OTA was remarkable (20–25 °C increases, Figure 3B), in agreement with the stronger crosslinking effect of OTA, as shown previously (Figure 1), with a more rigid film matrix (Figure 2). With both OFA and OTA, the enthalpy of transition for peak two also decreased from 111 J/g (control) to 86–108 J/g and 97–102 J/g, respectively, further suggesting a less ordered structure with the combined effect of the C–N or C–S covalent bonds in these phenol-crosslinked protein films. The temperature range of the thermal scan in the present study encompasses the temperatures applied to whey protein film sealing under an appropriate jaw pressure, e.g., 296 or 445 kPa [41] and 293 kPa [44]. It is not clear how the oxidized phenolic treatments would affect the sealability of WPI; this should be investigated in future research.

### 3.7. Protein Leachability

**Figure 4:**
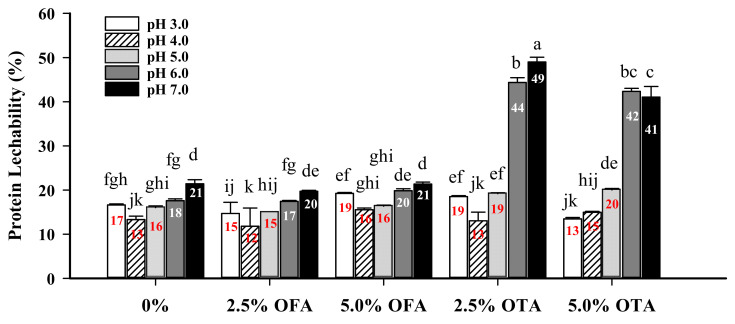
Protein leachability of 2.5% and 5.0% oxidized ferulic (OFA) and tannic (OTA) acid-treated WPI films at different pH levels. a–k: means without a common letter differ significantly (*p* < 0.05). Numbers inside the bars denote mean specific values of film protein leachability.

As an edible packaging material, protein-based films are digestible and hydratable in aqueous solutions. Unlike synthetic plastic polymer films, protein films are formed primarily through non-covalent interactions, although covalent bonds can be introduced through polyphenol crosslinking. The analysis of proteins that can readily leach into aqueous solutions can provide important guidance for practical applications [3,5]. As shown in Figure 4, the leaching rate of the control film at pH 3.0–7.0 was 13–21%. The leaching rates of the films formed with 2.5% and 5% OFA-crosslinked WPI were similar to the control, over the same pH range. However, while showing no difference from the control and OFA-treated films at pH 3.0–5.0, the protein leachability of the OTA-crosslinked films more than doubled at pH 6.0 and 7.0 when compared with other films. This high degree of protein leaching may be explained by the porosity of the OTA-crosslinked films (Figure 2). Due to the strong aggregation caused by OTA crosslinking, a heterogeneous structure, instead of an interactive, continuous, and well-connected WPI film matrix, would be conducive to protein leaching. The inter-polypeptide electrostatic repulsive forces at pH 6.0 and 7.0 would facilitate protein release from the film matrix.

### 3.8. Digestibility

In vitro pepsin→pancreatin sequential digestion was carried out to monitor the digestion behavior of WPI films, and elucidate the oxidized phenol crosslinking effect. As summarized in Table 4, generally, the digestibility increased with time, reaching 85.6% for the control and 65.9–91.0% for the phenol-treated film samples. However, the effects of phenols were noticeably variable; they slightly suppressed the WPI digestion by pepsin, but promoted the digestion by pancreatin upon extended incubation (180–240 min), except for the 5% OTA-treated film. The 5% OFA- and OTA-treated films were generally more digestible than 2.5% by pancreatin. It has been reported that unmodified whey protein components were weakly susceptible to pepsin cleavage [45,46]. The decreased digestibility for most phenol-treated films was likely caused by the chemical bonds formed in the films (e.g., C–N and C–S) that were unrecognizable by the enzyme. The slight temporary decline in the measured digestion rate, from 30 to 60 min, of pepsin incubation may be the result of the hydrophobic aggregation of partially hydrolyzed/unfolded βLg, as such phenomena have been observed by a number of other researchers [47,48].

## 4. Conclusions

This study demonstrates that oxidized ferulic and tannic acids were able to improve the film-forming properties of WPI, through the oxidative modification of protein side-chain groups and the aggregation behavior. However, the performance of OFA and OTA differed. OFA increased the film pull strength (TS) and elasticity (Young’s modulus), only at the higher treatment concentration (5.0%), but improved the film extensibility (EAB) at both the 2.5% and 5.0% concentrations. In contrast, OTA greatly increased the pull strength and elasticity at both the 2.5% and 5.0% concentrations, but did not affect the film extensibility. The degree of oxidative changes in WPI, particularly covalent crosslinking, attributed to the changed film properties. The increased size of the protein aggregates and changed protein network structure accounted for, at least in part, the modified film properties. Such whey protein-based films were hydratable and remained largely digestible, despite the slight inhibition by OTA. These key findings strongly suggest the possibility to fabricate tunable and functionality-enhanced whey protein films. However, when aiming at the specific mechanical property(ies) that are desired to achieve, care must be taken to generate an appropriate level and type of protein oxidative modification with phenolic compounds. This would allow the balanced protein crosslinking in developing the target structural characteristics of the protein film. It is conceivable that equally or more functional oxidizable phenolic acids than OFA or OTA exist, and possibly also flavonoids, which, when oxidized, will promote protein film formation. Further research is warranted to evaluate the practical application of such biodegradable films for the packaging and preservation of dry or semi-dry food materials and products.

## Figures and Tables

**Table 1:** Physicochemical changes in oxidized ferulic (OFA) and tannic (OTA) acid-treated WPI.

Parameter	Control	OFA (*w/w*)		OTA (%, *w/w*)	
		2.5%	5.0%	2.5%	5.0%
SH (nmol/mg protein)	21.8 ± 0.3 ^a^	16.4 ± 0.5 ^b^	11.4 ± 0.2 ^c^	4.2 ± 0.2 ^d^	1.2 ± 1.5 ^e^
NH_2_ (μmol/mg protein)	0.79 ± 0.02 ^a^	0.75 ± 0.02 ^b^	0.72 ± 0.01 ^b^	0.62 ± 0.01 ^c^	0.59 ± 0.01 ^d^
Hydrophobicity	2.59 ± 0.16 ^a^	2.62 ± 0.07 ^a^	2.48 ± 0.07 ^a^	1.94 ± 0.02 ^b^	1.72 ± 0.05 ^c^
Particle size (nm)	85 ± 9 ^a,b^	78 ± 1 ^b^	83 ± 6 ^b^	117 ± 18 ^a^	112 ± 48 ^a^
*ζ*-potential (mV)	−26.6 ± 1.9 ^a^	−26.5 ± 2.2 ^a^	−25.5 ± 1.8 ^a^	−24.8 ± 1.7 ^a^	−25.6 ± 2.4 ^a^

^a–e^ Means ± SD (*n* = 3) in the same row that do not share a common superscript differ significantly (*p* < 0.05). SH: sulfhydryls; NH_2_: free amines.

**Table 2:** Mechanical and selective physical properties of oxidized ferulic (OFA) and tannic (OTA) acid-treated WPI films.

Property	Control	OFA (*w/w*)		OTA (%, *w/w*)	
		2.5%	5.0%	2.5%	5.0%
TS (MPa)	4.5 ± 0.2 ^c^	4.9 ± 0.7 ^b,c^	5.2 ± 0.5 ^b^	6.7 ± 0.3 ^a^	6.7 ± 0.40 ^a^
EAB (%)	24.2 ± 4.1 ^b^	34.9 ± 10.0 ^a^	35.5 ± 11.9 ^a^	20.5 ± 4.3 ^b^	15.2 ± 1.8 ^b^
Young’s modulus (MPa)	190 ± 11 ^c^	215 ± 22 ^b,c^	238 ± 26 ^b^	358 ± 19 ^a^	376 ± 4 ^a^
Swelling	139 ± 61 ^b^	171 ± 48 ^b^	193 ± 7 ^b^	325 ± 21 ^a^	318 ± 86 ^a^
WVP (g·mm/kPa·h·m^2^)	1.02 ± 0.21 ^a^	1.00 ± 0.01 ^a^	0.94 ± 0.01 ^a^	0.95 ± 0.04 ^a^	0.95 ± 0.11 ^a^

^a–c^ Means ± SD (*n* = 3) in the same row that do not share a common superscript differ significantly (*p* < 0.05). TS: tensile strength; EAB: elongation at break; WVP: water vapor permeability.

**Table 3:** Light transmittance and transparency of oxidized ferulic (OFA) and tannic (OTA) acid-treated WPI films.

Treatment	Light Transmittance (%) at Different Wavelengths (nm)							Transparency
	200	280	350	400	500	600	800	
0%	0	0	70.7 ± 2.0 ^a^	78.5 ± 1.8 ^a^	82.9 ± 1.7 ^a^	85.0 ± 1.6 ^a^	89.0 ± 1.6 ^a^	1.0 ± 0.1 ^c^
2.5% OFA	0	0	12.7 ± 0.5 ^b^	67.1 ± 3.7 ^b^	80.2 ± 4.4 ^a^	83.0 ± 4.2 ^a^	87.7 ± 3.7 ^a^	1.1 ± 0.3 ^c^
5.0% OFA	0	0	0.4 ± 0.3 ^e^	44.9 ± 5.1 ^c^	80.5 ± 0.4 ^a^	84.1 ± 0.5 ^a^	88.8 ± 0.3 ^a^	1.1 ± 0.0 ^c^
2.5% OTA	0	0	8.9 ± 0.9 ^c^	29.7 ± 1.8 ^d^	58.4 ± 2.3 ^b^	69.2 ± 2.6 ^b^	77.5 ± 2.6 ^b^	2.7 ± 0.3 ^b^
5.0% OTA	0	0	2.4 ± 0.7 ^d^	14.7 ± 2.8 ^e^	45.0 ± 4.7 ^c^	59.3 ± 4.9 ^c^	69.9 ± 4.1 ^c^	4.5 ± 0.7 ^a^

^a–c^ Means ± SD (*n* = 3) in the same column that do not share a common superscript differ significantly (*p* < 0.05).

**Table 4:** In vitro sequential digestion rate of oxidized ferulic (OFA) and tannic (OTA) acid-treated WPI films ^1^.

Digestion	Treatment	Hydrolysis Time (min)						
		Pepsin			Pancreatin			
		0 ^2^	30	60	90	120	180	240
4% Pepsin (1 h 37 °C, pH 2.0)	0%	(35.2 ^a^)	56.0 ± 1.4 ^a^	44.8 ± 0.6 ^a,b^	56.0 ± 6.2 ^a^	72.7 ± 3.7 ^b^	70.0 ± 2.6 ^c^	85.8 ± 0.6 ^b^
	2.5% OFA	(32.3 ^a,b^)	47.1 ± 0.3 ^b^	44.0 ± 0.3 ^a,b^	48.0 ± 2.8 ^b^	62.2 ± 0.9 ^c^	81.1 ± 0.3 ^b^	91.0 ± 4.6 ^a^
	5.0% OFA	(29.0 ^b^)	44.1 ± 0.3 ^c^	46.6 ± 3.1 ^a^	40.7 ± 2.1 ^c^	57.9 ± 0.7 ^c,d^	80.0 ± 2.1 ^b^	82.4 ± 2.8 ^b^
↓								
4% Pancreatin (4 h, 37 °C, pH 7.5)	2.5% OTA	(27.6 ^b^)	49.3 ± 1.4 ^a,b^	42.4 ± 0.4 ^b^	54.7 ± 1.9 ^a^	77.2 ± 2.2 ^a^	89.0 ± 2.2 ^a^	89.1 ± 0.4 ^a^
	5.0% OTA	(28.5 ^b^)	40.7 ± 0.4 ^d^	28.4 ± 0.4 ^c^	34.4 ± 1.4 ^d^	60.0 ± 2.1 ^c^	62.1 ± 2.8 ^d^	65.9 ± 0.4 ^c^

^1^ Digestion rate: % of enzyme-released protein (into supernatant) over the total amount of unleached protein in the film. ^2^ Time “0” values in parentheses denote unleached protein over total protein in the original film (%), which were the base for digestion rate calculation. ^a–d^ Means ± SD (*n* = 3) in the same column that do not share a common superscript differ significantly (*p* < 0.05).

## Data Availability

The data presented in this study are as described in the individual figures and tables.
